# The research progress on the role of glucose-6-phosphate dehydrogenase in immune regulation

**DOI:** 10.7717/peerj.20971

**Published:** 2026-03-19

**Authors:** Dingmei Zhang, Yizhong Wang

**Affiliations:** 1 Affiliated Hospital of Zunyi Medical University, ZunYi, China; 2 Honghui Hospital of Xi’an Jiaotong University, Xi’an, China

**Keywords:** Glucose-6-phosphate dehydrogenase, Immune regulation, Immune cells, Immune-related diseases, Therapeutic target

## Abstract

Glucose-6-phosphate dehydrogenase (G6PD), the rate-limiting enzyme of the pentose phosphate pathway (PPP), plays a pivotal role in immune regulation by regulating metabolic reprogramming and redox homeostasis of immune cells. It mediates the production of nicotinamide adenine dinucleotide phosphate (NADPH) and ribose-5-phosphate (R5P), which are essential for the activation, proliferation, and effector function of T lymphocytes, B lymphocytes, macrophages, and neutrophils—specifically promoting T/B cell-mediated adaptive immunity and macrophage/neutrophil-mediated innate immune responses. Abnormal G6PD activity (deficiency or overexpression) is closely associated with the pathogenesis of immune-related diseases: G6PD deficiency increases susceptibility to autoimmune diseases (*e.g.*, rheumatoid arthritis, systemic lupus erythematosus) and infectious diseases (*e.g.*, hepatitis, malaria, COVID-19) by inducing oxidative stress and immune cell dysfunction; in tumor immunity, G6PD dualistically promotes tumor cell proliferation while regulating anti-tumor immunity *via* modulating cytoxic D8^+^ T cell exhaustion and macrophage polarization. Additionally, G6PD-targeted immunotherapies, including small-molecule inhibitors and gene therapy, have shown promising preclinical potential for treating immune-related diseases. These findings highlight G6PD as a key metabolic-immune hub, providing critical theoretical basis for understanding immune regulation mechanisms and developing novel diagnostic and therapeutic strategies for autoimmune diseases, infectious diseases, and tumors.

## Introduction

Glucose-6-phosphate dehydrogenase (G6PD), as a rate-limiting enzyme of the pentose phosphate pathway (PPP), plays a central role in cell metabolism (Battistuzzi et al., 1985; Uhlen et al., 2015). Reduced nicotinamide adenine dinucleotide phosphate (NADPH) and ribose-5-phosphate (R5P) catalyzed by it are very important to maintain intracellular redox homeostasis and support biosynthetic reaction. In red blood cells, G6PD deficiency leads to hemolytic anemia. Accumulating evidence indicates that G6PD participates in metabolic reprogramming, functional regulation and immune response regulation of immune cells, and its dysfunction is associated with tumor immunity and immune-related diseases, including increased susceptibility to infections, development of autoimmune diseases (*e.g.*, rheumatoid arthritis), and tumor immune escape (Shah et al., 2024; Yen et al., 2020). In recent years, increasing studies have focused on the role of G6PD in the field of immune regulation. For example, G6PD deficiency damages the NOX/p38 MAPK/AP-1 redox signaling pathway, leading to defective inflammasome activation and reduced bacterial clearance capacity, suggesting that G6PD affects innate immune response by regulating redox homeostasis (Yen et al., 2020). In-depth exploration of the relationship between G6PD and immune regulation will reveal the pathogenesis of immune-related diseases and provide theoretical basis for developing new diagnosis and treatment strategies.

## Survey Methodology

This review analyzed relevant literature published from 1985–2025, retrieved from PubMed (https://pubmed.ncbi.nlm.nih.gov/) and Web of Science (https://www.webofscience.com/). The search strategy combined main terms and subordinate terms with Boolean operators (AND, OR). Main terms: “G6PD”, “glucose-6-phosphate dehydrogenase”, “pentose phosphate pathway (PPP)”. Subordinate terms: “immune regulation”, “immune cells”, “T lymphocytes”, “B lymphocytes”, “macrophages”, “neutrophils”, “autoimmune diseases”, “infectious diseases”, “tumor immunity”, “immunotherapy”, “NADPH”, “reactive oxygen species (ROS)”. Example search string (PubMed): (G6PD OR “glucose-6-phosphate dehydrogenase”) AND (“immune regulation” OR “immune cells” OR “autoimmune diseases” OR “tumor immunity”). This review excluded editorials, letters sent to the editor, and case reports.

## Structure, Function and Regulation of G6PD

Understanding the gene and protein structure of G6PD is foundational to elucidating its enzymatic function and regulatory mechanisms, as structural features determine substrate binding, coenzyme specificity, and polymerization state—all key to its role in immune cell metabolism

### Gene and protein structure of G6PD

**Figure 1:**
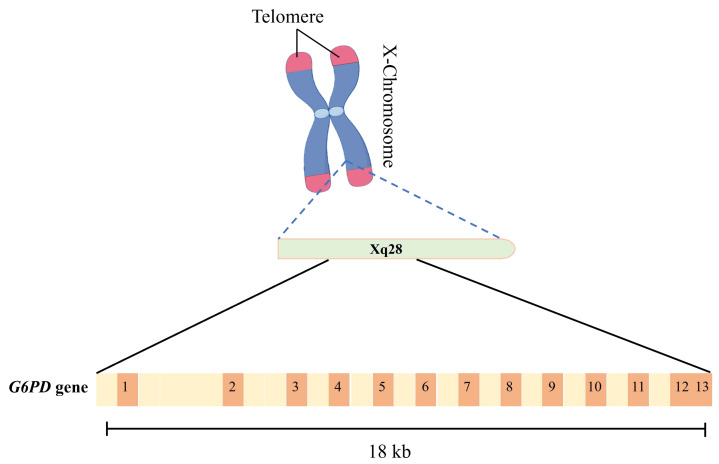
Diagram of G6PD gene. The *G6PD* gene is located in the long arm 2 region 8 band (Xq28) of human X chromosome, with a total length of about 18kb, including 13 exons and 12 introns.

The *G6PD* gene is located in the long arm two region eight band (Xq28) of human X chromosome, with a total length of about 18 kb, including 13 exons and 12 introns (Fig. 1). G6PD protein consists of 515 amino acid residues with a molecular weight of about 59 kDa. It has two key domains: N-terminal rossmann folded domain and C-terminal helical domain. Rossmann folded domain is responsible for binding substrate glucose-6-phosphate (G6P) and coenzyme NADP^+^, in which conserved amino acid residues play a decisive role in substrate specificity and enzyme activity; C-terminal helix domain is involved in protein dimerization and stability maintenance (Au et al., 2000; Cappellini & Fiorelli, 2008; Kotaka et al., 2005; Stanton, 2012). G6PD exists *in vivo* as a dimer or tetramer under physiological conditions: the dimer serves as the basal catalytic form with intrinsic activity, while tetramers typically exhibit significantly higher catalytic efficiency due to conformational stabilization mediated by the C-terminal helical domain (Kotaka et al., 2005; Luzzatto, 2006; Qu et al., 2025).

### The central role of G6PD in the pentose phosphate pathway

**Figure 2:**
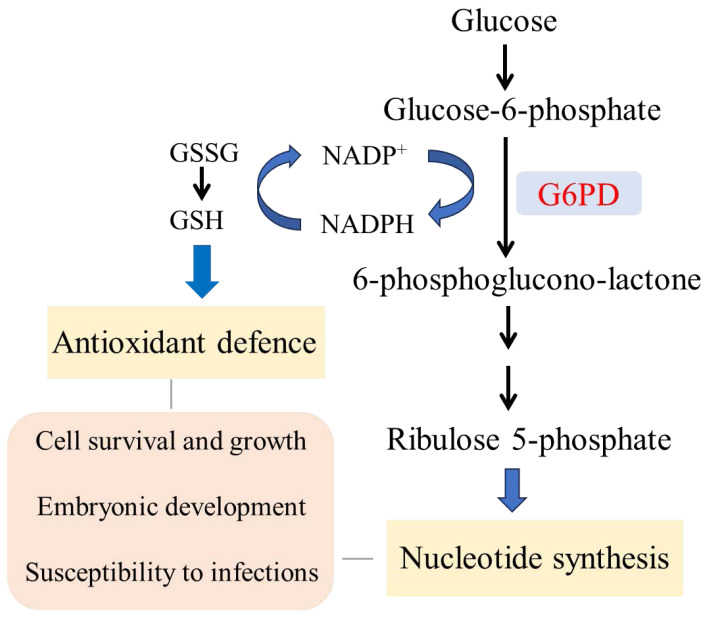
The function of glucose-6-phosphate dehydrogenase (G6PD). G6PD catalyzes PPP to produce nicotinamide adenine dinucleotidephosphate (NADPH), an essential cofactor for glutathione (GSH) regeneration, and ribose-5-phosphate (R5P), an essential substrate for nucleotide synthesis. Therefore, G6PD plays an important role in cell survival and growth, embryonic development, viral infections and so on.

PPP can be divided into an oxidative phase and a non-oxidative phase. As the rate-limiting enzyme of the oxidative phase, G6PD determines the metabolic flux of the PPP. In the oxidative phase, G6P is converted to 6-phosphogluconolactone under the catalysis of G6PD, with NADP^+^ reduced to NADPH; 6-phosphogluconolactone is further hydrolyzed to 6-phosphogluconate, which is then decarboxylated by 6-phosphogluconate dehydrogenase (6-PGD) to generate NADPH, CO_2_, and R5P (Fig. 2) (Stanton, 2012). NADPH has diverse cellular functions: on the one hand, it participates in biosynthetic reactions (*e.g.*, fatty acid, cholesterol, and nucleotide synthesis) to provide a material basis for cell growth and proliferation; on the other hand, it maintains the reduced state of glutathione (GSH) and participates in scavenging reactive oxygen species (ROS) to protect cells from oxidative damage. R5P, an important raw material for nucleotide synthesis, is involved in DNA and RNA synthesis.

### Multi-level regulation mechanism of G6PD activity

G6PD activity is regulated at multiple levels, including transcriptional regulation, post-translational modification, and metabolite feedback regulation (Meng et al., 2022). At the transcription level, nuclear factor-κB (*NF-*κ*B*) and signal transduction and activator of transcription 3 (*STAT3*) can bind to the promoter region of *G6PD* gene, promoting its transcription under inflammatory stimulation or oxidative stress. In terms of post-translational modification, G6PD can undergo phosphorylation, acetylation, glycosylation and ubiquitination. Phosphorylation of G6PD exhibits dual effects depending on the kinase involved. For example, protein kinase A (PKA) phosphorylates serine of G6PD, resulting in a decrease in its enzymatic activity (Xu, Osborne & Stanton, 2005); in contrast, protein kinase C (PKC)-mediated phosphorylation at S210 and T266 enhances the catalytic activity of G6PD (Gupte et al., 2011). Acetylation predominantly inhibits G6PD activity through modifications at key functional sites (K403, K235, K171), while only acetylation at K89 activates G6PD (Ai et al., 2016; Wang et al., 2014; Wu et al., 2023). This site-specific regulation underscores the complexity of acetylation as a post-translational modification in tuning metabolic enzyme function. In metabolite feedback regulation, increased NADPH/NADP ratio can inhibit G6PD activity to avoid excessive accumulation (Stanton, 2012).

## Metabolism and Functional Regulation of G6PD in Immune cells

**Figure 3:**
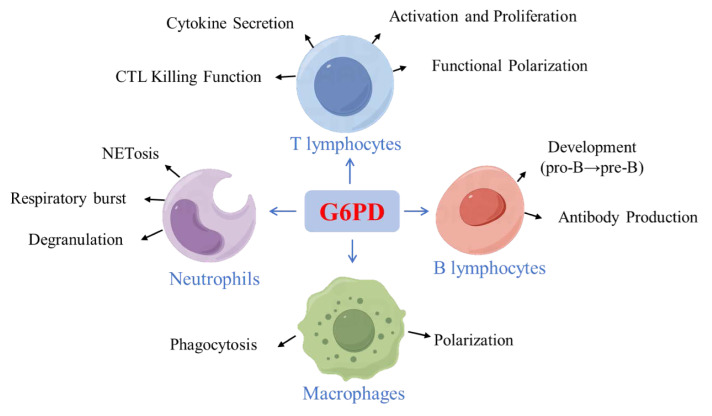
Schematic diagram of the regulatory network of G6PD on immune cell function. G6PD can regulate the activation, differentiation and effector functions of T cells, B cells, macrophages and neutrophils by controlling the level of NADPH. Image credit: Figdraw software.

PPP plays a key role in the immune system, such as the production of cytokines and oxidative bursts, which affect the state and function of immune cells. Different types of immune cells have unique metabolic demands: phagocytic immune cells (*e.g.*, macrophages and neutrophils) kill pathogens through oxidative bursts, while T cells require extensive biosynthesis and energy to proliferate, activate, and secrete cytokines. Thus, the PPP plays distinct roles in different cell types, and G6PD, as the rate-limiting enzyme of the PPP, also plays an important role in immune regulation (Fig. 3) (TeSlaa et al., 2023).

### T lymphocytes

#### Effect of G6PD on T cell activation and proliferation

The activation and proliferation of T cells is the key link of adaptive immune response, which is accompanied by significant changes in cell metabolism (Ma et al., 2024). Resting T cells mainly rely on oxidative phosphorylation to produce energy for basic metabolic needs. Upon antigen stimulation, T cells rapidly initiate metabolic reprogramming, with glycolysis and PPP activity significantly enhanced to provide sufficient energy and biosynthetic raw materials for activation, proliferation, and function (Kishton, Sukumar & Restifo, 2017). G6PD plays an indispensable role in this process: at the initial stage of T cell activation, G6PD activity is rapidly upregulated, and large amounts of NADPH are produced through the PPP to meet the demand for reducing equivalents during activation (Gu et al., 2021; Shah et al., 2024). NADPH not only participates in maintaining redox homeostasis in cells and preventing oxidative stress damage but also provides reducing power for the synthesis of biofilm components such as fatty acids and cholesterol, and supports the proliferation of T cells and the expansion of cell membrane. Inhibition of G6PD activity will lead to the decrease of NADPH level and the increase of oxidative stress in T cells, and then inhibit the activation and proliferation of T cells (Shah et al., 2024) (Fig. 3). *In vitro* experiments, after treating T cells with G6PD specific inhibitors, the proliferation response of T cells stimulated by antigen was obviously weakened, the cell cycle process was hindered, and the survival rate was reduced, indicating that G6PD is a key enzyme necessary for T cell activation and proliferation (Daneshmandi et al., 2021; Ghergurovich et al., 2020). However, in T cells from patients with rheumatoid arthritis (RA), upregulated G6PD expression leads to NADPH accumulation and ROS consumption, while impaired redox signaling induces T cell hyperproliferation and inflammatory factor production (Yang et al., 2016).

#### G6PD regulates cytokine secretion and functional polarization of T cells

**Figure 4:**
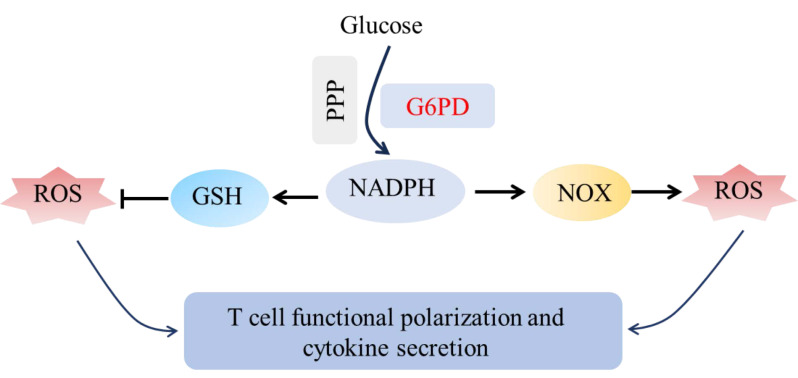
Schematic diagram of G6PD regulating T cells *via* the NADPH-ROS axis. G6PD drives NADPH production: NADPH fuels NOX to generate ROS, while supporting GSH-mediated ROS clearance. Tuned ROS levels govern T cell functional polarization and cytokine secretion.

After activation, T cells can differentiate into different functional subsets, such as helper T cells (Th; a subset of CD4+ T cells, *i,e.*, Th1, Th2, and Th17) and regulatory T cells (Treg), *etc.* Each subset plays different immunomodulatory functions by secreting specific cytokines (Vignali, Barbi & Pan, 2017). Moderate ROS is very important for T cell survival, proliferation and Th1 differentiation (such as IFN-γ and TNF-α production), but excessive ROS will lead to Th1 overactivation and inflammatory disorder (Franchina, Dostert & Brenner, 2018). G6PD maintains glutathione (GSH) reduction by producing NADPH, thus eliminating excessive ROS, which plays an important role in regulating T cell functional polarization and cytokine secretion (Fig. 4). For example, in T lymphoblastic leukemia, G6PD is down-regulated by inhibiting mTOR, which leads to ROS accumulation and cell death, suggesting the protective effect of G6PD in T cell hypermetabolism (Silic-Benussi et al., 2022). However, in T cells from RA patients, G6PD overexpression leads to excessive ROS consumption, promoting T cell differentiation into pro-inflammatory Th1 and Th17 cells and exacerbating inflammation (Fig. 4) (Yang et al., 2016).

Treg cells are critical for maintaining immune tolerance and homeostasis. A study using bioinformatics analysis and experimental verification identified *G6PD* as one of the key Treg-related genes associated with Treg function in pulmonary hypertension (PH), which is significantly downregulated in the lung tissues of severe PH patients and hypoxia-induced PH mice (Liu et al., 2022). The study indicates that G6PD may participate in the progression of PH by regulating Treg-mediated immune and inflammatory responses. Given the importance of the PPP in the immunosuppressive function of Treg cells (TeSlaa et al., 2023), the regulatory mechanisms of G6PD-mediated PPP activity on Treg cell function can be elaborated as follows: First, G6PD promotes NADPH production *via* the PPP, maintains the redox homeostasis of Treg cells, and thereby prevents Treg cells from functional impairment induced by oxidative stress. Second, G6PD-mediated PPP activity may regulate the production of immunosuppressive cytokines by Treg cells. Therefore, it is speculated that G6PD activity is also strictly regulated in Treg cells, affecting their survival, proliferation, and immunosuppressive function (Fig. 3).

#### Role in cytotoxic T lymphocytes killing function

CD8^+^ T cells are also known as cytotoxic T lymphocytes (CTL), which are a type of cells expressed with the CD8 subset on their surface. CTLs are key effector cells that directly kill target cells in adaptive immune responses, with their killing function relying on secreted cytotoxic substances (*e.g.*, perforin and granzyme) and cytokines (*e.g.*, IFN-γ). Studies have found that tumor cells can downregulate G6PD expression by inducing H3K9me3 deposition in the *G6PD* promoter, inhibiting the PPP, reducing acetyl-CoA production, and decreasing H3K9 acetylation in the granzyme B (Gzmb) promoter, leading to CTL exhaustion. Activating G6PD can enhance CTL killing activity and inhibit tumor growth (Lu et al., 2022). By regulating PPP metabolism and ROS homeostasis, G6PD acts as a key metabolic checkpoint in T cells. Additionally, G6PD interacts with lactate dehydrogenase B (LDHB), which restricts G6PD dimerization (a prerequisite for its catalytic activation), thereby reducing NADPH production and triggering disulfidptosis-dependent CD8+ T cell exhaustion. Heterozygous loss of G6PD not only promotes disulfidptosis but also enhances ferroptosis in CD8+ T cells, further exacerbating their exhaustion and impairing antitumor immunity (Wan et al., 2025). As a key hub connecting CD8+ T cell metabolism and function, G6PD’s future exploration in T cell memory and immune balance will expand metabolism-immunity cross-regulation insights, offering novel targets for immune-related diseases and shifting the therapeutic paradigm toward metabolic-immune network modulation.

### G6PD participates in B lymphocytes development and antibody production

The development process of B lymphocytes includes a series of stages: differentiation from hematopoietic stem cells to mature B cells, and further differentiation into plasma cells to produce antibodies and memory B cells under the stimulation of antigens, or formation of germinal center (GC). This process requires precise metabolic regulation, in which G6PD plays an important role (Fig. 3). In bone marrow, during the transition from pro-B cells to pre-B cells, transcription factors paired box protein 5 (PAX5) and Ikaros family zinc finger protein 1 (IKZF1) downregulate glycolytic enzymes (including G6PD) by inhibiting the PI3K-AKT pathway, forcing B cells into a hypometabolic state that relies on AMPK to maintain energy balance (Chan et al., 2017). This “energy deprivation” state prevents abnormal proliferation of B cells and ensures directional differentiation of lineage, which is of great significance to the early development stage of B cells in bone marrow. Mature B cells have high PPP activity, supporting their constitutive antibody secretion and rapid immune responses (Martinis et al., 2025). Other studies have also found that in resting B cells, G6PD expression is inhibited by transcription factors PAX5 and IKZF1, resulting in low basal activity. However, B cell activation depends on upregulated G6PD and PPP activity to cope with oxidative stress, highlighting the importance of G6PD in normal B cell development and tumorigenesis (Xiao et al., 2018). Upon activation by B cell receptors (BCR) or Toll-like receptors (TLR), B cells use the PI3K-AKT-mTOR pathway to drive glucose uptake, with part of the glucose entering the PPP *via* G6PD to produce NADPH and R5P, supporting antioxidant defense and nucleotide synthesis (Martinis et al., 2025). Long-lived plasma cells (LLPCs) and long-lived memory B cells derived from GCs can quickly respond to secondary antigen challenges, providing long-term protection against specific antigens (Martinis et al., 2025). In GC reactions, GC B cells (GCBCs) are highly dependent on NADPH produced by the PPP to maintain redox balance (Brookens et al., 2024); knocking down *G6PD* (*e.g.*, *via* shRNA interference) hinders GCBC differentiation, reduces class switching, and inhibits antibody production (Wu et al., 2025). These studies reveal the key role of G6PD in B cell development and antibody production.

### Macrophages

#### G6PD regulates macrophages polarization and function

Macrophages, an important component of innate immunity, are highly plastic and can polarize into distinct functional phenotypes (mainly classically activated M1 macrophages and alternatively activated M2 macrophages) in response to microenvironmental signals, exerting different roles in immune and inflammatory responses (Zhang et al., 2023). G6PD is involved in the metabolic regulation of macrophage polarization (Zamani, Hoseini & Namin, 2019) (Fig. 3). In studies by Parsanathan & Jain (2021) when G6PD-deficient monocytes polarize into macrophages, the expression of M1 surface markers (CD64 and CD80) and M2 surface markers (CD11B and CD209) increases, with more significant changes in CD80 and CD209 expression, indicating that G6PD deficiency activates monocytes, induces their polarization, and makes macrophages exhibit M2-like polarization characteristics. In hypoxia-induced lung inflammation, G6PD inhibitors reduce the expression of M2a macrophage markers (Arg1, CD163), suggesting that G6PD may promote macrophage polarization toward the M2a phenotype, participating in chronic inflammation and tissue repair (Hashimoto & Gupte, 2022). In terms of functional cytokine gene expression, G6PD deficiency increases the expression of pro-inflammatory markers (*e.g.*, TNF and MCP-1) while decreasing the level of pro-fibrotic or M2 functional cytokines (*e.g.*, TGF-β), indicating that G6PD deficiency promotes the transformation of macrophage function toward pro-inflammation and affects its pro-fibrotic functions (Parsanathan & Jain, 2021). In triple-negative breast cancer (TNBC) cells, G6PD overexpression promotes the secretion of CCL2 and TGF-β1, drives M2 macrophage polarization, and accelerates tumor metastasis (Li et al., 2023a). In a hypoxic pulmonary hypertension model, G6PD can also induce pro-inflammatory epigenetic signals (including upregulating pro-inflammatory factors such as TNF-α), increase the number of activated macrophages, activate platelets, and drive immunopathological vascular diseases (Hashimoto & Gupte, 2022). These studies show that regulating G6PD activity can modulate macrophage polarization, affect immune and inflammatory responses, and provide potential targets for treating inflammation-related diseases.

#### Mechanism of G6PD in macrophage phagocytosis and bactericidal function

Phagocytosis and killing of pathogens by macrophages depend on ROS produced by respiratory burst (Forman & Torres, 2002). The G6PD-involved PPP provides the NADPH necessary for the respiratory burst (also known as oxidative burst): upon recognizing and phagocytosing pathogens, macrophages activate NADPH oxidase (NOX), which uses NADPH as a substrate to produce ROS (*e.g.*, superoxide anion (O_2_^−^) and H_2_O_2_) that exert bactericidal effects in phagolysosomes (Wang et al., 2023; Weigert et al., 2018). On one hand, G6PD promotes the production of ROS by increasing the generation of NADPH to serve as a substrate for NOX, a key enzyme in the respiratory burst. Ultimately, it influences the anti-mycobacterium function of macrophages by enhancing the respiratory burst (Rana et al., 2024). On the other hand, NADPH generated by the PPP catalyzed by G6PD serves as a critical cofactor for inducible nitric oxide synthase (iNOS) to catalyze the conversion of arginine to nitric oxide (NO), thereby regulating the ability of macrophages to kill the amastigotes of *Leishmania major* (Zamani, Hoseini & Namin, 2019). Additionally, G6PD may also affect the fusion of phagocytes and lysosomes, pathogen clearance and other processes by regulating other metabolic pathways and signaling pathways in macrophages. For example, in the research on obesity, macrophage G6PD promotes ROS/RNS production by producing NADPH, activates p38 MAPK and NF-kB pathway, and induces the expression of pro-inflammatory factors (*e.g.*, IL-6 and IL-1β), thereby regulating the pro-inflammatory response of macrophages (Ham et al., 2013).

### Neutrophils

Neutrophils, the most abundant leukocytes in circulating blood, play a central role in pathogen surveillance and tissue damage responses. Upon stimulation by pathogen components (*e.g.*, chemokines, cytokines, lipopolysaccharides) or injured tissue signals, neutrophils rapidly migrate to injury and infection sites and initiate innate immune responses, accompanied by rapid metabolic pattern transformation, in which G6PD plays a decisive role (Ghergurovich et al., 2020) (Fig. 3). Within minutes of initial stimulation, neutrophils switch from a glycolysis-based basal metabolic mode to the PPP, essentially driven by an urgent need for NADPH (Britt et al., 2022). As the rate-limiting enzyme of the PPP, G6PD activity directly determines the rate of NADPH production, thereby affecting the execution of multiple neutrophil effector functions. It has been found that NADPH oxidase function in granulocytes is significantly impaired in patients with severe G6PD deficiency, leading to defective neutrophil extracellular trap (NET) formation (Siler et al., 2017).

At the level of immune defense mechanism, the rapid production of NADPH supports three core functions of neutrophils: providing electron donors for the explosive synthesis of ROS in phagolysosomes (ROS, as key antibacterial substances, can effectively kill phagocytosed pathogens); second, driving the release of NETs, which form an antibacterial network *via* extracellular release of chromatin and granular proteins to limit pathogen spread; third, promoting granule exocytosis (degranulation) accompanied by massive extracellular ROS production, enhancing the local antibacterial microenvironment (Azevedo et al., 2015). Notably, the NETosis process is highly dependent on superoxide anions produced by NADPH oxidase. G6PD not only accelerates ROS production by promoting PPP flux but also directly promotes NET formation by oxidizing and inhibiting glyceraldehyde-3-phosphate dehydrogenase (GAPDH) activity(Leben et al., 2018; Talwar et al., 2023).

However, this G6PD-dependent metabolic regulation has a double-edged sword effect. In severe infections (*e.g.*, cytokine storms caused by SARS-CoV-2 infection), overactivated PPP and ROS production may lead to pathological NETosis, exacerbating tissue damage and inflammatory runaway (Li et al., 2023b). This suggests that G6PD-mediated metabolic reprogramming is not only a core mechanism for neutrophils to rapidly respond to infections but also a key node linking immune defense and immunopathology. In-depth analysis of the dynamic regulatory network of G6PD in neutrophils will help reveal the metabolic basis of innate immune responses and provide potential intervention targets for targeted therapy of infectious and autoimmune diseases.

## Association Between Abnormal G6PD and Immune-Related Diseases

### Autoimmune diseases

**Figure 5:**
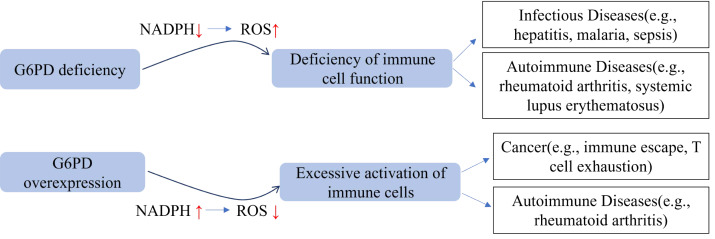
Diagram illustrates the association between G6PD abnormalities and immune-related diseases. Abnormal G6PD activity (deficiency or overexpression) is associated with infectious diseases, autoimmune diseases, and tumors by disrupting the metabolism and function of immune cells.

Numerous studies have shown that G6PD deficiency is associated with an increased risk of autoimmune diseases (Ding et al., 2024; Dore, Fanciulli & Pes, 2023; Israel et al., 2023) (Fig. 5). A retrospective cohort study in Israel, including 7,473 G6PD-deficient patients and 29,892 matched controls, found that the incidence of autoimmune diseases (*e.g.*, rheumatoid arthritis (RA), systemic lupus erythematosus (SLE), and scleroderma) was significantly higher in G6PD-deficient patients (Israel et al., 2023). A recent study has shown that G6PD is significantly upregulated in patients with RA, suggesting that its upregulated expression enhances the activity of the PPP (Golestanifar et al., 2025). On the one hand, it maintains the oxidative homeostasis of immune cells and prevents their apoptosis; on the other hand, it supports the metabolic reprogramming and proliferation of immune cells, ultimately promoting chronic inflammatory responses. Meanwhile, as a potential diagnostic biomarker, it possesses both pathological regulatory functions and clinical application value, making it a key molecule linking immunity and metabolism in RA. However, in previous studies, G6PD expression was found to be downregulated in erythrocyte of patients with RA (Gheita et al., 2014). Although there are discrepancies in G6PD expression across different cell types in RA patients, the essence lies in cell type-specific regulation driven by differences in cellular functions and pathological demands. Specifically, G6PD is decreased in erythrocyte due to exhaustion induced by oxidative stress, whereas it is upregulated in peripheral blood mononuclear cells (PBMCs) in response to metabolic reprogramming and inflammatory activation of immune cells (Gheita et al., 2014; Golestanifar et al., 2025). While these two observations appear contradictory, they essentially reflect the core pathological features of RA—chronic oxidative stress and immunometabolic dysregulation, which representing adaptive responses of distinct cell populations during the same disease process. These findings highlight the context-dependent role of G6PD in RA pathogenesis and underscore the necessity of cell-type-specific analyses for advancing our understanding of disease mechanisms and developing precise diagnostic and therapeutic strategies.

The deficiency of G6PD activity is one of the key factors driving the immune disorder in SLE. Compared with RA, the role of G6PD in SLE is more focused on immune disorders mediated by redox imbalance, red blood cell damage and target organ involvement (Chang et al., 2026; Israel et al., 2023; Liang et al., 2025), as oxidative stress is a potential causal factor for SLE (Fujii et al., 2015). Its abnormal activity (deficiency or variation) participates in the pathogenesis and disease progression of SLE through multiple pathways, with the core mechanism revolving around the abnormal immune cell function and red blood cell metabolic disorders.

Scleroderma, also known as systemic sclerosis (SSc), has a pathogenesis involving vascular pathology, immune dysfunction, fibrosis, and the interactions among these three processes. Patients with scleroderma exhibit a significantly enhanced state of oxidative stress, and excessive ROS can induce vascular endothelial injury, promote fibroblast activation, and lead to excessive collagen deposition (Vona et al., 2018; Zhang et al., 2022). As a core antioxidant enzyme, the key role of G6PD in scleroderma may focus on regulating redox balance, thereby affecting vascular endothelial function, immune cell activity, and fibroblast phenotypic transition. Although studies have confirmed that G6PD deficiency is associated with a significant increase in the risk of SSc (Israel et al., 2023), the specific molecular mechanisms by which G6PD deficiency participates in the processes of vascular pathology, immune dysregulation, and fibrosis in SSc through disrupting redox balance have not yet been systematically elucidated.

In conclusion, the mechanism by which G6PD deficiency leads to autoimmune diseases may be related to oxidative stress and abnormal immune cell function (Fujii et al., 2015). G6PD deficiency increases intracellular oxidative stress levels, and excessive ROS damages DNA, proteins, and lipids, producing new antigens that activate the immune system and trigger autoimmune responses.

### Infectious diseases

Severe *G6PD* mutations can impair enzyme catalytic capacity, resulting in immunodeficiency (Fig. 5) (Vives Corrons et al., 1982), making G6PD-deficient patients more susceptible to certain infectious diseases. For example, G6PD deficiency is associated with increased susceptibility to hepatitis A and E viruses (Ahmad et al., 2018; Miri-Aliabad, Khajeh & Shahraki, 2017). In malaria infection, G6PD-deficient erythrocytes have weakened antioxidant capacity and are more susceptible to Plasmodium infection. After infection, Plasmodium growth and reproduction in erythrocytes are affected, leading to different clinical symptoms and disease progression compared to non-G6PD-deficient individuals (Luzzatto, Nannelli & Notaro, 2016; Taleb et al., 2025). In other bacterial and viral infections, G6PD-deficient patients may have reduced infection resistance due to abnormal immune cell function, with mechanisms possibly involving impaired immune cell metabolism, changes in erythrocyte surface molecules, and effects of hemolytic products on the immune microenvironment (Bouguerra et al., 2021; Shah et al., 2024; Sun et al., 2022; Yen et al., 2020). Severe G6PD deficiency have more severe inflammatory reaction and higher risk of sepsis (TeSlaa et al., 2023). In COVID-19, G6PD deficiency can worsen the disease by aggravating oxidative stress and pro-inflammatory signals (*e.g.*, TNF-α, IL-6), while antioxidant therapy (*e.g.*, vitamin C, N-acetylcysteine) and anti-aging drugs (*e.g.*, metformin) may improve the prognosis by regulating G6PD-related pathways (Yang et al., 2021).

### Tumor immunity

G6PD plays a complex role in tumor immunity. On the one hand, the activity of G6PD in tumor cells is up-regulated, and a large number of NADPH and R5P are produced through PPP, which meets the needs of rapid proliferation of tumor cells and resistance to oxidative stress, and promotes tumor growth (Ahamed et al., 2023). On the other hand, the function of G6PD in immune cells affects tumor immune response (Fig. 5). For example, G6PD cooperates with NF-κ B-induced kinase (NIK) to enhance the metabolic adaptability and effector function of T cells, thereby inhibiting tumor growth (Gu et al., 2021). A study on hepatocellular carcinoma (HCC) found that knocking down *G6PD* enhances CD8^+^ T cell proliferation and IFN-γ secretion, while overexpression inhibits T cell function. It was further confirmed that G6PD affects the expression of PD-L1 by regulating mTOR pathway, inhibiting the activation of CD8^+^ T cells and promoting tumor immune escape (Qu et al., 2025). Recent studies have found that inhibiting G6PD activity can lead to NADPH depletion in CD8^+^ T cells, trigger disulfidptosis, accelerate T cell exhaustion, and weaken anti-tumor immunity (Wan et al., 2025). It has been pointed out that G6PD is a metabolic checkpoint for tumors to activate CTLs, and enhancing G6PD activity or PPP metabolism in CTLs can improve their ability to kill tumor cells (Lu et al., 2022). Additionally, regulating G6PD activity in macrophages and affecting their polarization direction can alter the immune state in the tumor microenvironment (Li et al., 2023a). These studies suggest that exploring the mechanism of G6PD in tumor immunity is helpful to develop new tumor immunotherapy strategies.

## Research Progress of Immunotherapy Targeting G6PD

In this section, we systematically reviewed the research progress of G6PD-targeted immunotherapy strategies (small molecule drugs, gene therapy, combined therapy), highlighting their potential clinical application value. This provides direction for future research and drug development.

### Small molecule drugs regulating G6PD activity

Developing small molecule drugs to regulate G6PD activity is one of the research directions of immunotherapy. Specific inhibitors targeting G6PD (which has high activity in tumor cells) can block PPP metabolism, reduce NADPH and R5P production, inhibit tumor cell proliferation, and enhance their sensitivity to oxidative stress. Meanwhile, for diseases with abnormal immune function caused by G6PD deficiency, developing drugs that enhance G6PD activity or compensate for NADPH deficiency is expected to improve immune function. A recently developed small-molecule G6PD inhibitor, G6PDi-1, can effectively inhibit G6PD activity, significantly reduce NADPH levels in immune cells (*e.g.*, T cells), inhibit inflammatory cytokine production in T cells, and reduce neutrophil respiratory bursts, indicating that G6PDi-1 can be used as a pharmacological target for regulating immune responses (Ghergurovich et al., 2020). Currently, some small-molecule drugs have entered preclinical research (see Table 1), but their specificity, safety, and efficacy require further verification.

**Table 1:** Summary of several G6PD small-molecule inhibitors.

Inhibitor	Inhibition type	Core mechanism	References
G6PDi-1	Reversible non-competitive inhibition	Directly binds to the allosteric site of *G6PD*, does not affect enzyme expression, specifically consumes NADPH in lymphocytes, and inhibits inflammatory factor production.	Ghergurovich et al. (2020)
Chrysomycin A (Chr-A)	Downregulating protein expression	Reduces G6PD protein expression (without affecting mRNA) through transcriptional regulation, and simultaneously downregulates metabolic enzymes such as HK2 and GLS, synergistically inhibiting glucose/glutamate metabolism.	Liu et al. (2024)
6-Aminonicotinamide (6-AN)	Competitive inhibition (against NADP^+^)	As a structural analog of NADP^+^, directly binds to the coenzyme-binding site of *G6PD*, blocking NADP^+^ binding to the enzyme and inhibiting the oxidative branch of PPP.	Li et al. (2023c)
Dehydroepiandrosterone (DHEA)	Non-competitive inhibition (partial downregulation of expression)	1. Non-competitively binds to G6PD, reducing V_max_; 2. Partially downregulates *G6PD* transcription; 3. Easily converted to steroid hormones *in vivo*, with non-specific effects.	Chanda et al. (2023)
Polydatin	Direct enzyme activity inhibition (non-competitive tendency)	Directly binds to G6PD protein, does not affect mRNA/protein expression, inhibits enzyme activity in a concentration-dependent manner, and induces ROS-mediated endoplasmic reticulum (ER) stress (IRE1/PERK pathway).	Mele et al. (2018)
Quercetin	Competitive inhibition (against NADP^+^-binding domain)	Binds to the NADP^+^-binding domain of *G6PD*, competitively blocks coenzyme binding, and induces oxidative damage to G6PD, impairing enzyme activity.	Ge et al. (2023)

### Gene therapy strategies

Gene therapy provides a new way to correct G6PD deficiency-related immune abnormalities. Through gene-editing technologies such as CRISPR-Cas9 system, the mutation of *G6PD* gene was repaired and the normal expression and activity of G6PD were restored (Tang et al., 2017). Alternatively, normal *G6PD* genes can be introduced into G6PD-deficient cells *via* gene vectors to improve cellular metabolism and immune function (Leopold et al., 2003; Mejias et al., 2006; Mondal et al., 2025). Gene therapy has shown effectiveness in animal model studies, but its safety, long-term efficacy, and ethical issues require further research and resolution.

### Combined treatment regimens

The combination of G6PD regulation and immune checkpoint inhibitors holds potential for tumor therapy. Preclinical studies have shown that inhibiting G6PD in tumor cells reduces their antioxidant capacity and proliferation (Chanda et al., 2023; Ghergurovich et al., 2020), while immune checkpoint inhibitors (*e.g.*, anti-PD-1) relieve T cell exhaustion (Budimir et al., 2022). Although direct evidence of synergistic effects is still limited, a recent study on HCC demonstrated that knocking down G6PD reduces the expression of PD-L1 therapy and increases CD8^+^ T cell infiltration and IFN-γ secretion (Qu et al., 2025). These studies suggested that G6PD modulation may optimize immune checkpoint inhibitor responses. Further clinical trials are needed to validate these synergistic effects. In the treatment of autoimmune diseases, the combination of regulating G6PD activity and immunomodulatory drugs may control immune response more effectively and improve disease symptoms.

By supplementing NADPH precursors (*e.g.*, glutamine, N-acetylcysteine) or R5P analogues, G6PD deficiency can be bypassed and the metabolic function of T cells can be restored. Preclinical studies have shown that glutamine supplementation can improve the proliferation and cytokine secretion of G6PD deficient T cells (Ryan & Tekwani, 2021; Zhong et al., 2021), which provides a new direction for immune support therapy of G6PD deficient patients.

## Conclusion and Prospect

G6PD plays a key role in immune regulation: it affects the intensity and direction of immune responses by participating in the metabolism and functional regulation of immune cells and is closely associated with the occurrence and development of many immune-related diseases. Immunotherapy based on G6PD target provides new ideas and strategies for the treatment of immune-related diseases. However, there are still many problems to be solved. In basic research, the specific molecular mechanism of G6PD in immune cell metabolism and function regulation, and its interaction with other metabolic pathways and signaling pathways still need further study. In clinical application, developing G6PD regulatory drugs and gene therapy methods with high specificity and safety, and optimizing combined therapy schemes are the focus of future research. The *in vivo* consequences of G6PD inhibition will also reflect its effects on other immune cell types, including inhibition of neutrophil oxidative bursts (Ghergurovich et al., 2020). With in-depth research, G6PD is expected to become an important target for the prevention and treatment of immune-related diseases, bringing new treatment hope for patients.

### Target audience

This review is primarily intended for four core groups of readers (basic research scientists, clinical researchers and practitioners, drug and gene therapy developers, graduate and doctoral students), aligned with its interdisciplinary focus on G6PD and immune regulation.

## Additional Information and Declarations

### Data Availability

The following information was supplied regarding data availability: This is a literature review.
